# Case Report: Complete and Fast Recovery From Severe COVID-19 in a Pemphigus Patient Treated With Rituximab

**DOI:** 10.3389/fimmu.2021.665522

**Published:** 2021-04-16

**Authors:** Jo Linda Sinagra, Claudio Vedovelli, Raffaella Binazzi, Adele Salemme, Francesco Moro, Cinzia Mazzanti, Biagio Didona, Giovanni Di Zenzo

**Affiliations:** ^1^ Molecular and Cell Biology Laboratory IDI-IRCCS, Rome, Italy; ^2^ Department of Infectious Diseases, Central Hospital of Bolzano, Bolzano, Italy; ^3^ Rare Diseases Unit, IDI-IRCCS, Rome, Italy

**Keywords:** pemphigus, autoimmune blistering disease, rituximab, immunosuppression, COVID-19, B cell

## Abstract

COVID-19 is characterized by a severe pulmonary disease due to severe acute respiratory syndrome (SARS)-CoV-2 infection. For clinicians involved in the management of patients with chronic autoimmune diseases the risk linked to the conditions itself and to drug-induced immunosuppression during the COVID-19 pandemic is a major topic. Pemphigus is a rare autoimmune blistering disease (AIBD) of the skin and mucous membranes caused by autoantibodies to desmosomal components, desmoglein 1 and 3. Among immunosuppressant therapies, rituximab (RTX) is considered a highly effective treatment with a favorable safety profile, but it induces a prolonged B-cell depletion that can lead to higher susceptibility to infections. For this reason, concerns about its use during the pandemic have been raised. We describe a case of a pemphigus patient in which RTX-induced B cell depletion led to the severe inflammatory phase, whereas corticosteroid treatment allowed a favorable outcome.

## Introduction

The novel coronavirus SARS-CoV-2 is the pathogenic agent responsible for COVID-19, a severe pulmonary disease, the dramatic worldwide outbreak of which led the WHO to announce a pandemic in March 2020 (1). Several aspects of COVID-19 epidemiology, transmission and morbidity in the general population are still unclear. Some mortality risk factors have been identified, such as older age, male sex and certain comorbidities (obesity, diabetes, heart disease, lung disease, kidney disease) (1, 2). There is some evidence that immunocompromised patients do not have a higher incidence or complications from COVID-19 than the general population (3–5). Despite this, for clinicians involved in the management of patients with chronic autoimmune diseases the risk linked to the conditions itself and to drug-induced immunosuppression is a major topic.

Pemphigus is an autoimmune blistering disease (AIBD) of the skin and mucous membranes in which autoantibodies targeting cell-cell adhesion molecules (desmoglein 1 and 3) induce blister formation. Standard pemphigus treatment includes corticosteroids and a variety of immunosuppressants (azathioprine, cyclophosphamide, mycophenolate) (6). Even if their use is not contraindicated during the pandemic, adjusting the dosage could be considered in patients with active COVID-19 infection (7). In this context the use of IV immunoglobulins appears to be of particular interest due to their role in immunity support and the absence of immunosuppressive effects (2, 8). Rituximab (RTX) an anti-CD20 monoclonal antibody that induces a prolonged B-cell depletion, is considered a highly effective treatment with a favorable safety profile and is included among the first line therapies for this disease (9–11). However, as it can lead to higher susceptibility to infections, concerns about its use during the pandemic have been raised (2). Some authors have suggested avoiding or temporarily postponing RTX during the pandemic due to the risk of SARS-CoV-2 infection (2, 12).

Few studies have been published about the clinical course of COVID-19 in pemphigus patients treated with RTX, and none have been published on patients infected immediately after RTX treatment.

We describe a case of a pemphigus patient with RTX-induced B-cell depletion who contracted COVID-19 with a severe course and had a favorable clinical outcome.

## Case-Report

We contacted 31 pemphigus patients routinely followed-up in our hospital in Rome who received RTX in the 12 months preceding the COVID-19 pandemic. Among them, a 45-year-old woman (professional nurse, living in Northern Italy) confirmed having COVID-19.

**Figure 1:**
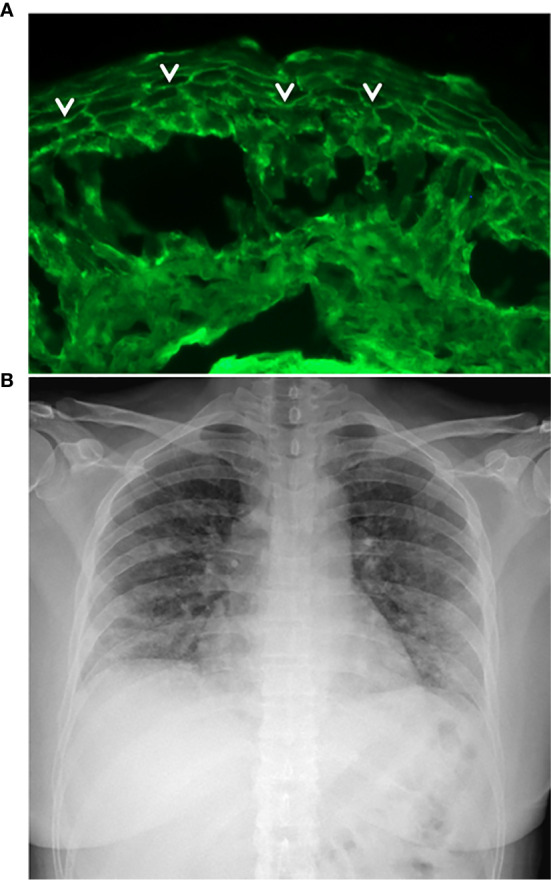
Essential elements for pemphigus and COVID-19 diagnosis. **(A)** Intercellular deposition of IgG (arrow heads) by direct immunofluorescence microscopy; **(B)** Chest radiograph of pemphigus patient with COVID-19 showing interstitial pneumonia with bilateral airspace opacities.

**Figure 2:**
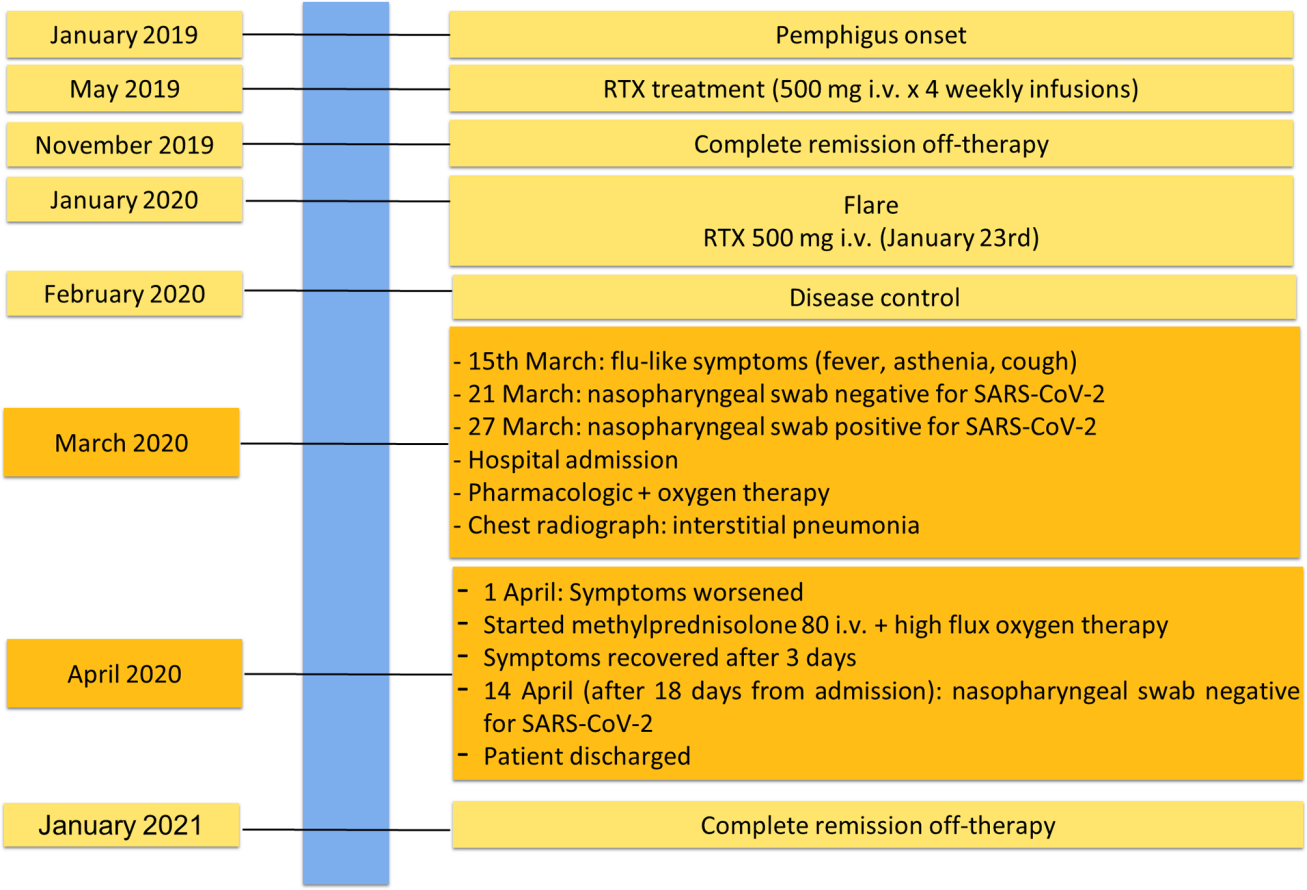
Timeline with relevant data from the episode of care.

She was affected by pemphigus foliaceous, as assessed by clinical examination and direct immunofluorescence microscopy (**Figure 1A**), from January 2019 and received a cycle of RTX (500 mg x 4 weekly infusions) in May 2019 reaching complete remission off-therapy in November 2019 (**Figure 2**). Two months later, a relapse occurred; therefore, on January 23 2020, she received an additional 500 mg RTX infusion and started prednisone 50 mg/day. Disease control was reached two weeks after infusion, prednisone was gradually tapered to 5 mg and lesions completely resolved at the beginning of March when B cells were still depleted. By the 15^th^ of March, she developed mild flu-like symptoms (fever up to 37.5°C, asthenia and some cough). She was treated with azithromycin 500 mg/day for 3 days without clinical improvement. On the 21^st^ of March, a nasopharyngeal swab was performed, resulting in negative SARS-CoV-2 result. She was started on Co-amoxiclav 875/125 mg bid for 6 days. During antibiotic treatment she experienced hyposmia, ageusia, dry cough, nocturnal dyspnea and hyperpyrexia (up to 39.5 C). As symptoms persisted, on March 27 a second nasopharyngeal swab was performed, resulting in positivity for SARS-CoV-2 (**Figure 2**). She was admitted to the Geriatric-COVID-19 Unit of the referral hospital in Bolzano-Bozen. At admission, the patient’s oxygen saturation was 94% on room air, ABG revealed pO2 61.9 mmHg, pCO2 34 mmHg, pO2(a)/FO2(l) 295 mmHg and pH 7.49. Laboratory tests showed lymphopenia (940 cells/µL, n.v. 1100-4500), slightly increased LDH (337 U/L, n.v. 123-230), increased CRP (11.03 mg/dl, n.v. < 0.05) and increased IL-6 (51.7 pg/ml, n.v. <7.0). The chest radiograph showed interstitial pneumonia with bilateral airspace opacities (**Figure 1B**). She was started on hydroxychloroquine (200 mg bid for 8 days), lopinavir/ritonavir (200/50 mg qd for 8 days) and enoxaparin 0.4 ml qd. Oxygen therapy (from 3 up to 8 l/min) was administered. Despite this, her fever persisted, dyspnea worsened and on April 1, she was transferred to the Infectious Diseases Unit of the same hospital. High flux oxygen therapy was given through a nonrebreather mask, intravenous methylprednisolone 80 mg qd was added and enoxaparin was increased to 0.4 ml bid, resulting in a sudden clinical improvement. Three days later, the patient was apyretic, her oxygen saturation improved to 98% with 4-6 liters of oxygen. Intravenous methylprednisolone was continued for 2 days and then gradually tapered to 16 mg per os. The patient’s conditions recovered. After two positive swabs, 18 days from admission a further nasopharyngeal swab was negative for SARS-CoV-2, so the patient was discharged continuing oral methylprednisolone 8 mg qd (**Figure 2**). At present (February 2021), the patient is in good general condition and her pemphigus is in complete remission off-therapy. She had no pulmonary sequelae and reported no difficulties in returning to her everyday habits or work.

## Discussion

Pemphigus patients can have a higher risk of infection due to exposed lesions and immunosuppressive treatments. As it is a rare disease, at present few cases of COVID-19 in RTX-treated pemphigus patients have been reported (7, 13). To our knowledge this is the first detailed case of severe COVID-19 in a pemphigus patient treated with RTX shortly before infection with a complete B cell depletion.

Shahidi-Dadras et al. reported 5 COVID-19 cases in a cohort of 45 RTX treated pemphigus patients, none of whom had a severe course. All patients received RTX at least one year before infection, thus they did not have a severe reduction in B cell count (14). Uzuncakmak et al. reported only one case of COVID-19 among 48 pemphigus patients treated with RTX during the past five years. The patient received RTX seven months before COVID-19 and had a mild course (15). Recently, Mahmoudi et al. reported 21 COVID-19 cases from a cohort of patients with AIBDs. Thirteen of them received RTX in the previous six months, but no specific information was provided about the temporal distance of COVID-19 infections from RTX and B cell counts (13).

COVID-19 seems to primarily involve T lymphocytes (16). As reported by Chen and coworkers, the number of CD4+ and CD8+ T cells was markedly lower in severe cases of COVID-19 than in moderate cases. In contrast, neither the proportion nor the number of B cells was reduced in most patients (16). From a histopathological point of view, in lung biopsy, the infiltrated lymphocytes were mostly CD3-positive (17). In animal models, similar to other coronaviruses, after comparing T cell–deficient mice and B cell–deficient mice Zhao et al. found that T cells are able to survive and kill virus-infected cells in the infected lung (18), highlighting the role of T lymphocytes in the pathogenesis and outcome of SARS-CoV and MERS-CoV infection. Niu et al. found that IgM/IgG and IgA B cell responses were induced in the early phase of infection (19). IgA may migrate to the respiratory tract, the gastrointestinal tract, or other mucosal sites to play an early immune function in virus clearance. In this context, the reduction of the B cell and/or T cell repertoire and their function, such as that which occurs in older patients, could limit viral clearance and prolong the innate proinflammatory response (20, 21). In particular, in severe patients an excessive inflammatory response induced by neutrophils and monocytes leads to potentially fatal hypercytokinemia (22). Zhang and coworkers identified several genes upregulated in severe patients that are responsible for the recruitment of neutrophils that probably represent the main contributor to disease progression (23). These authors also demonstrated a downregulation of the type I interferon (IFN) response, especially in severe patients (23). This phenomenon could impair the first line of defense against viral infection exerted by IFNs and reduce their ability to limit the overactive inflammatory response leading the patient to a more severe course (23). In our patient, RTX-induced B cell depletion may have impaired the immune response, preventing a rapid virus clearance. Thereafter, as described in a subgroup of patients (24), an exaggerated immune response led to inflammatory-induced lung injury and worsening of the disease. The severe disease course of our 46-year-old female patient appears quite different from the reported general disease course for her sex and age-range (25). In fact, a correlation between COVID-19 disease severity and age and sex has been recently suggested, with older age and male sex being associated with more severe disease (25, 26). An Italian study on 1591 patients admitted to intensive care units showed that only 143/1591 (8.9%) were in the 41-50 age range, and they were predominantly males (27). Moreover, pulmonary involvement seems to be more severe and prolonged in patients over 60 years of age (28). However, when inflammatory-induced lung injury occurs, immunosuppressants, such as steroids, might be useful in suppressing inflammation. In this regard, a randomized trial from Oxford University (RECOVERY) investigated the role of several drugs (lopinavir-ritonavir, hydroxychloroquine, corticosteroids, azithromycin, convalescent plasma or tocilizumab) on mortality reduction in COVID-19 patients (29). The researchers found that in 2104 patients receiving dexamethasone 6 mg once per day for ten days vs 4321 patients on usual care, dexamethasone reduced deaths by one-third in ventilated patients and by one-fifth in other patients receiving oxygen only (30). Moreover, the REMAP CAP trial demonstrated that high dosages of intravenous hydrocortisone (50 mg or 100 mg every 6 hours or shock-dependent dosage) vs no hydrocortisone treatment in patients with severe COVID-19 were associated with 93% and 80% probabilities of better clinical outcome (31). In line with these data in our patient, the use of endovenous methylprednisolone resulted in a sudden clinical improvement, confirming the efficacy of this drug in controlling the inflammatory phase.

Different viral agents are associated with an increased risk of more severe disease course and respiratory complications in immunocompromised patients (18, 19). However, during different coronavirus outbreaks, such as SARS and Middle East respiratory syndrome no increased mortality was reported in immunosuppressed transplanted patients, or those affected by cancer or autoimmune diseases (1, 19). Preliminary findings on patients with chronic arthritis treated with immunosuppressive therapy do not suggest an increased risk of respiratory or life-threatening complications from SARS-CoV-2 (3).

During the COVID-19 pandemic, experts and dermatologic society opinions were divided about the obvious need to counterpart the risks and benefits of continuing or suspending immunosuppressive treatment (32). In particular, several controversies have emerged about the use of RTX with pemphigus patients. According to some authors, anti-CD20 therapies do not seem to necessarily imply higher rates of infection and risk for more severe COVID-19 disease (7). However, the cited systematic review from Kasperkiewicz and coworkers did not include pemphigus patients treated with RTX shortly before infection as in our patient (33). In contrast, in line with our findings Mahmoudi et al. found that in 13 patients with AIBDs who contracted COVID-19 after receiving RTX during the preceding 6 months, the risk of hospitalization decreased each month after RTX (13).

In conclusion, in our patient with a RTX-induced B cell depletion and inflammatory-induced lung injury, methylprednisolone treatment controlled the inflammatory phase, resulting in sudden clinical improvement. In addition, as suggested by Mahmoudi and coworkers, in our patient RTX-induced B cell depletion might be a risk factor for exacerbation of COVID-19 (13). Thus, as data are still scarce and as patients treated with anti-CD20 drugs are in general at higher risk for infections, the use of RTX during the pandemic has to be carefully pondered on a case-by-case basis (2, 7). Moreover, in the view of the recent development of COVID-19 vaccines, RTX treatment could be preferably considered at least 4 weeks after a complete vaccine cycle (34).

Due to the rarity of autoimmune blistering diseases, we think that also anecdotal cases could also contribute to improving the therapeutic management of these patients during the pandemic.

## Data Availability Statement

Clinical and laboratory data of patient are stored in Bolzano Hospital archives. Requests to access these datasets should be directed to Dr. Raffella Binazzi, Raffaella.binazzi@sabes.it.

## Ethics Statement

Written informed consent was obtained from the individual(s) for the publication of any potentially identifiable images or data included in this article.

## Author Contributions

JS contributed to writing and editing of the manuscript. JS and GZ contributed to conception. JS, CV, RB, CM, FM, and AS contributed to data collecting. GZ and BD made the supervision. All authors contributed to the article and approved the submitted version.

## Funding

This article was supported by the “Progetto Ricerca Corrente - 2020” of the Italian Ministry of Health, Rome, Italy.

## Conflict of Interest

The authors declare that the research was conducted in the absence of any commercial or financial relationships that could be construed as a potential conflict of interest.

## Abbreviations

RTX, rituximab; AIBD, autoimmune blistering disease; SARS, severe acute respiratory syndrome.
